# A hybrid automated treatment planning solution for esophageal cancer

**DOI:** 10.1186/s13014-019-1443-5

**Published:** 2019-12-19

**Authors:** Chifang Ling, Xu Han, Peng Zhai, Hao Xu, Jiayan Chen, Jiazhou Wang, Weigang Hu

**Affiliations:** 1 Department of Radiation Oncology, Fudan University Shanghai Cancer Center, Shanghai, 200032 China; 2 Department of Oncology, Shanghai Medical College, Fudan University, 270 Dongan Road, Shanghai, 200032 China

**Keywords:** Automated planning, Knowledge-based planning, Esophageal carcinoma

## Abstract

**Objective:**

This study aims to investigate a hybrid automated treatment planning (HAP) solution that combines knowledge-based planning (KBP) and script-based planning for esophageal cancer.

**Methods:**

In order to fully investigate the advantages of HAP, three planning strategies were implemented in the present study: HAP, KBP, and full manual planning. Each method was applied to 20 patients. For HAP and KBP, the objective functions for plan optimization were generated from a dose–volume histogram (DVH) estimation model, which was based on 70 esophageal patients. Script-based automated planning was used for HAP, while the regular IMRT inverse planning method was used for KBP. For full manual planning, clinical standards were applied to create the plans. Paired *t*-tests were performed to compare the differences in dose-volume indices among the three planning methods.

**Results:**

Among the three planning strategies, HAP exhibited the best performance in all dose-volume indices, except for PTV dose homogeneity and lung V5. PTV conformity and spinal cord sparing were significantly improved in HAP (*P* < 0.001). Compared to KBP, HAP improved all indices, except for lung V5. Furthermore, the OAR sparing and target coverage between HAP and full manual planning were similar. Moreover, HAP had the shortest average planning time (57 min), when compared to KBP (63 min) and full manual planning (118 min).

**Conclusion:**

HAP is an effective planning strategy for obtaining a high quality treatment plan for esophageal cancer.

## Introduction

Esophageal cancer is one of the most common thoracic malignancies, but more than 60% of patients are at a relatively late stage when diagnosed, resulting in non-eligibility for surgical resection. Radiotherapy is one of the standard options for advanced/late stage cancer [1]. Since 1985, an increasing number of patients have undergone preoperative radiotherapy to downstage tumor, and achieved higher cure rates [2]. Beginning in 2001, the prevalence of intensity modulated radiation therapy (IMRT) has led to better organs at risk (OAR) protection without compromising tumor coverage, when compared to three-dimensional conformal radiation therapy (3DCRT) [2].

At present, pursuing optimal plans remains a time-consuming and demanding task, especially for less experienced physicists/dosimetrists. Typically, plan optimization requires planners to adjust plan parameters according to the difference between current dose distribution and clinical goals. Common parameters involved are beam orientation, normalization, optimization objectives and their priorities/weights. This trial-and-error process could take a few hours, or sometimes, a few days [3]. The recently emergence of automated planning techniques has improved the overall treatment plan quality, consistency and planning efficiency [4–6].

There are two major types of automated planning techniques: script-based planning and knowledge-based planning (KBP) [7].

Script-based planning follows the general planning strategies and steps that experienced planners usually take during optimization. Its automation relies on the optimization algorithm itself, without taking individual anatomy, prior planning experience into account.

The KBP is another approach that requires a plan library. It utilizes statistical models, which are developed with plan libraries, to predict achievable dose-volume histograms (DVH), then automatically generates optimization objectives given predicted DVHs, patient and beam geometry. However, it relies on the classic, user dependent optimization process to generate the final dose map.

The present study introduces a hybrid automated treatment planning (HAP) solution that combines different mechanisms of the two approaches mentioned above. The performance of HAP was evaluated by comparing its results with KBP and manual planning (MP).

## Methods

**Fig. 1:**
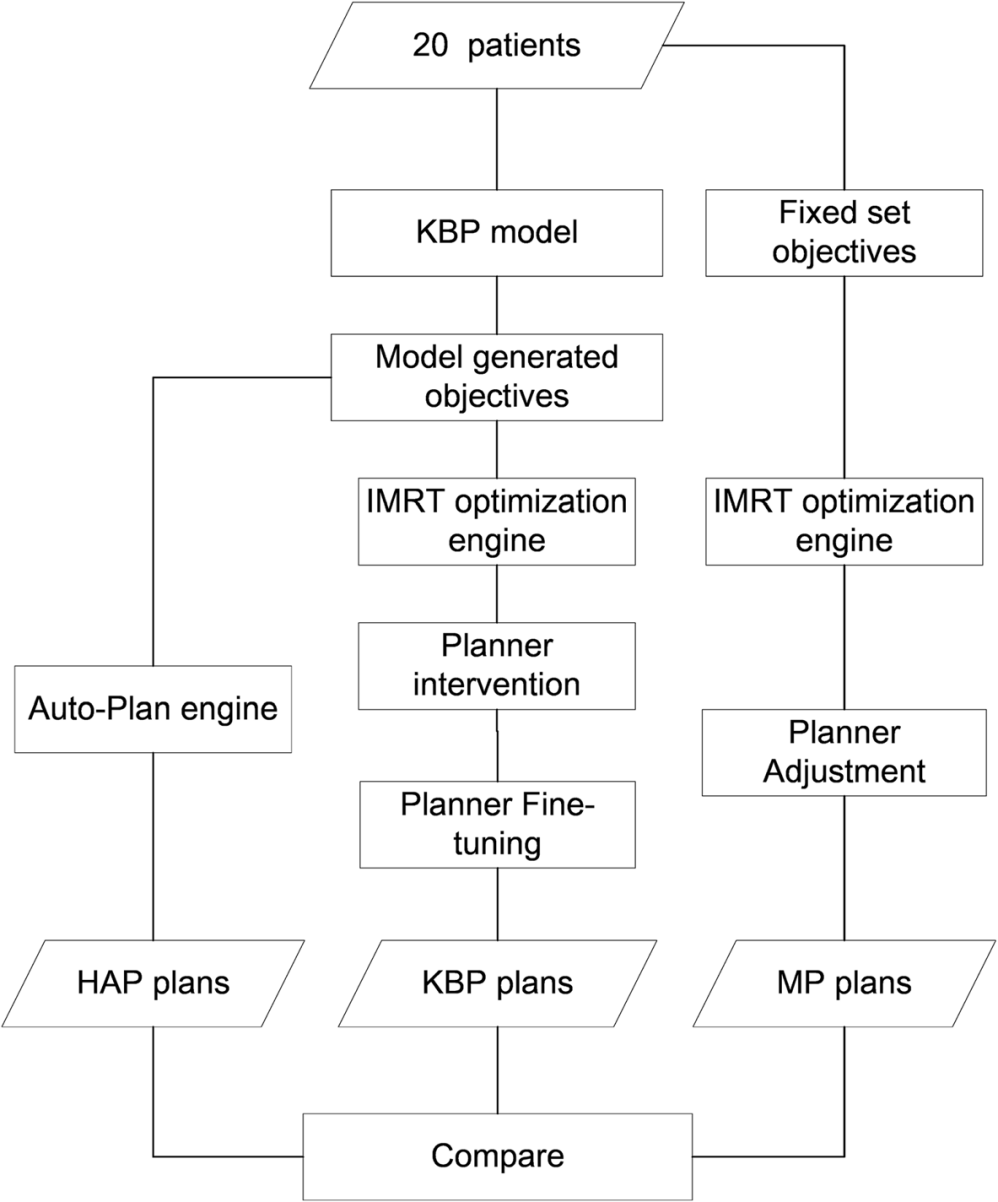
Workflow of the present study. *Abbreviations:* KBP = knowledge-based planning; IMRT = intensity modulated radiation therapy; HAP = hybrid automated planning; MP = manual planning

To fully compare these three planning approaches, three sets of plans were created: (1) Manual plans, (2) KBP plans and (3) HAP plans. Details of how these plans were developed can be found in section 2.3, 2.4 and 2.5 respectively, and the general work flow is illustrated in Fig. 1. Statistical analysis of the comparison were described in section 2.7. For the purpose of this study, a Varian Trilogy Linac model and fixed gantry IMRT technique with 6MV energy were chosen for all plans. Same beam configuration was applied to each patient’s all three types of plans. All optimizing work were done in Pinnacle 9.10 (Philips Medical System, Fitchburg, WI, USA) while Eclipse 13.5 Varian Medical System, Palo Alto, CA) was only used to generate planning objectives. One dosimetrist with 3 years’ experience performed all the planning work and a senior oncologist having more than 10 years’ experience in radiotherapy for thoracic region reviewed all the plans.

### Patients

A total of 20 patients with esophageal cancer treated in the department of radiation oncology between June 2016 and June 2018 were included in this study. The patient characteristics are summarized in Table 1.

**Table 1:** Clinical features of the 20 patients with thoracic esophageal cancer

Characteristics	No. of patients
PTV length (cm)	
< 14	8
14–18	4
> 18	8
PTV volume (cc)	
> 400	5
300–400	10
< 300	5
Gender	
Male	12
Female	8
Age (year)	40–60
Lung volume (cc)	
2000–3000	4
3000–4000	10
> 4000	6

### Target volume and OAR delineation

All patients were scanned in supine position during simulation. A chest board was used to rest their arms over their heads. Clinical target volume (CTV) and planning target volume (PTV) as well as bilateral lungs, heart and spinal cord were contoured on Planning CT (3 mm slice thickness). The lung-PTV structure defined as bilateral lungs minus PTV, was used for optimization and monitoring lung dose.

### Manual plans

20 plans were designed following departmental planning protocol. Each plan consisted of 5–7 coplanar beams. Gantry angles were determined by the dosimetrist considering the position of PTV in relation to OARs. The dose prescribed to PTV was 61.2Gy in 34 fractions, with a fractional dose of 1.8Gy. A pre-defined template containing objectives that were moderately tighter than the dose constraints listed in the protocol, was used for initial optimization. Subsequent cycles of optimization involving adding dose control structures (e.g. rings, planning organ at risk volumes), adjusting objectives or their weights, finetuning dose distribution, were at the planner’s discretion. Planning goals were including prescribed dose covering at least 95% PTV, maximum dose not exceeding 110% of prescription dose, all OARs’ dose constraints being met.

### KBP plans

RadpidPlan is Varian’s commercial solution of knowledge-based DVH prediction algorithm. It incorporates geometric and associated dosimetric information extracted from existing clinical plans into a statistical model called principle component analysis (PCA). Given beam orientation, it estimates the range of achievable DVH taking into account patient specific anatomy, and automatically generates objectives based on estimated DVHs [8, 9].

To create KBP plans, firstly, a new PCA model was trained with 70 previously delivered plans. Then, by applying this model to the each selected patient, predicted DVHs were generated for lung-PTV, heart and spinal cord, and their corresponding objectives (Table 2 column 2 and 3 excluding those for ring structures) were manually entered into Pinnacle’s optimization algorithm. After that, the planner proceeded to initial optimization with identical beam arrangement of MP and followed general planning steps described in previous section.

**Table 2:** Setting of the objective dose and constraints in the hybrid auto-planning (HAP) and KBP-only planning

OARs	Objective functions	Weighting (KBP-only)	Weighting (HAP)
Lung-PTV	Max DVH V20	50	Medium
	Max DVH V30	50	Medium
	Max DVH V5	50	Medium
	Max EUD	30	Low
Heart	Max DVH V30	50	Medium
	Max EUD	30	Low
Spinal cord	Max dose	100	Medium
SC + 0.3	Max dose	100	Medium
Ring 1	Max dose	50	Medium
Ring 2	Mad dose	50	Medium
Ring 3	Max dose	30	Low

Note: SC + 0.3 represents the volume around the spinal cord with a 3 mm radius

Ring 1 represents the volume around PTV with a 5 mm radius

Ring 2 represents the volume around PTV with a 10 mm radius

Ring 3 represents the volume around PTV with a 15 mm radius

### HAP plans

Auto-Planning is an integrated module in Pinnacle 9.10. Its concept originated from reginal optimization [10], then was implemented based on the regions of interest (ROIs) [11] and now has been matured as an automated optimization engine [8]. Auto-Planning mimics the decision-making process of an experienced planner with a progressive optimization algorithm which continually adjusts planning objectives based on the difference between planning goals set by the user and current DVH parameters. In addition to that, Auto-Planning automates optimizing process by automatically adding planning structures and objectives for general planning tasks such as managing targets uniformity and conformity, sparing OARs, controlling dose fall-off outside targets [12]. In some cases, Auto-Planning could dramatically reduce the number of objectives required for optimization [13].

Similar to KBP plans, planning objectives were inherited from model prediction, but inputted into Auto-Planning engine instead. Beam configurations were identical to MP and KBP. For PTV, only the prescription dose needs to be entered, as target dose uniformity and conformity are automatically controlled by a built-in module with preset parameters. Four priority levels are available for OARs: low, medium, high, and constraint. The objectives and their associated weights used for HAP plans are presented in Table column 2 and 4. Unlike MP or KBP, no further human interventions other than normalization adjustment were performed for HAP plans after the initial optimization. Then plan was taken as it was out of a single run.

### Evaluation metrics

Metrics used for plan evaluation and comparison are listed in the first column in Table 3. Homogeneity index (HI) was defined as

$$ \mathrm{HI}=\frac{\mathrm{D}1\%-\mathrm{D}99\%}{\mathrm{D}50} $$

**Table 3:** PTV and OAR sparing comparison

	Mean ± SD			*P*-value		
	KBP-only	HAP	Manual	KBP-only vs. HAP	KBP-only vs. Manual	HAP vs. Manual
PTV						
HI	0.13 ± 0.03	0.12 ± 0.02	0.11 ± 0.02	0.04	< 0.001	0.11
CI	0.64 ± 0.05	0.74 ± 0.03	0.69 ± 0.04	< 0.001	< 0.001	< 0.001
D_1%_	67.42 ± 1.42	67.08 ± 1.05	66.39 ± 0.98	0.15	< 0.001	0.02
D_99%_	58.98 ± 0.58	59.36 ± 0.46	59.2 ± 0.61	0.01	0.11	0.24
Lung-PTV						
V_5_	47.85 ± 18.66	52.00 ± 18.40	47.71 ± 13.61	0.1	0.11	0.01
V_20_	22.55 ± 6.91	20.16 ± 6.14	21.30 ± 5.90	0.01	0.22	0.07
V_30_	11.63 ± 4.11	10.19 ± 4.05	12.22 ± 4.55	< 0.001	0.25	< 0.001
D_mean_	11.45 ± 3.24	11.09 ± 3.42	11.37 ± 3.14	0.01	0.78	0.33
Heart						
V_30_	21.27 ± 17.05	19.12 ± 16.47	22.35 ± 18.66	0.02	0.40	< 0.001
V_40_	13.89 ± 12.07	11.81 ± 11.12	14.97 ± 13.04	0.01	0.37	0.01
D_mean_	14.76 ± 10.74	14.05 ± 10.49	15.32 ± 11.47	0.05	0.25	< 0.001
Spinal Cord						
D_max_	43.99 ± 2.00	41.42 ± 2.60	43.61 ± 2.2	< 0.001	< 0.001	< 0.001
Planning time (range)	62 min (15–122)	57 min (20–106)	118 min (22–210)	0.42	< 0.001	< 0.001

Where Dx% is the dose received by x% volume. Conformity index (CI) was defines as

$$ \mathrm{CI}=\frac{\mathrm{V}95\%\ast \mathrm{V}95\%}{\mathrm{PIV}\ast \mathrm{TV}}; $$

Where PIV is the total volume covered by 95% of the prescription dose, TV is the volume of the PTV, and V95% is the volume of the PTV covered by 95% of the prescription dose.

### Statistical analysis

Paired *t*-tests were performed to compare the differences among the three planning strategies. The SPSS statistical software (IBM, Chicago, IL, USA) was used for the analysis.

## Results

The overall plan quality of the three planning strategies were similar, whereas HAP and KBP took much shorter time than MP (*P* < 0.001). Table 3 presents the results of the comparison of all dose metrics and planning time. Approved by the senior oncologist, all plans were clinically acceptable.

**Fig. 2:**
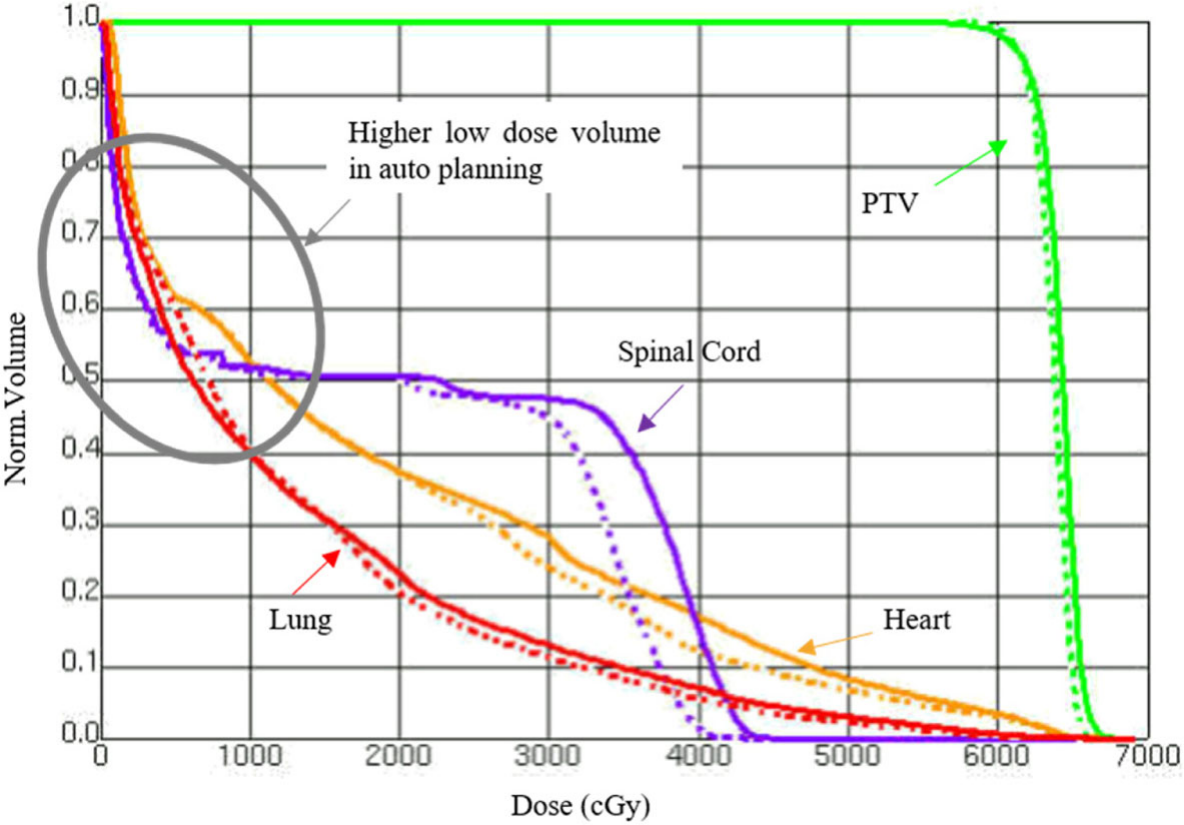
The dose distribution in the transverse slice

**Fig. 3:**
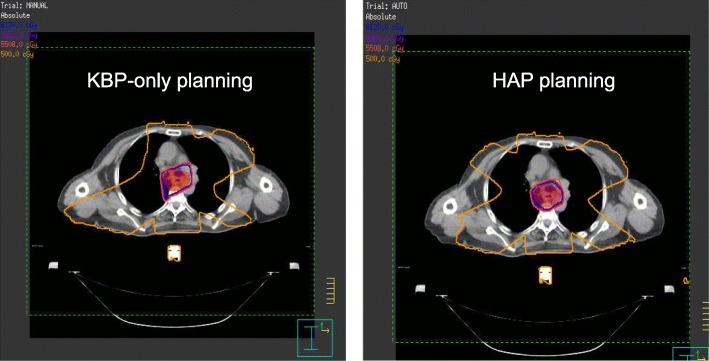
Dose-volume histogram for the two strategies for the large target area PTV and OARs (the solid line represents the KBP strategy, while the dashed line represents the HAP strategy)

HAP achieved significantly better CI than KPB and MP (*P* < 0.001). Figure 2 shows the difference of dose distribution in KBP and HAP. Regarding OAR protection, the heart and the spinal cord in HAP group received less dose than those in the other two groups. Also except for V5, the mean values of dose metrics for lung were lowest with HAP, even though for most dose metrics the differences did not reach statistical significance. A representative DVH comparison between KBP and HAP is shown in Fig. 3.

## Discussion

The present study combined two different automation mechanisms (knowledge-based and scripts-based) for esophageal cancer planning. This method greatly lessens a physicist’s planning time, because it does not require the iterative modification of objectives to pursue the optimal plans for esophageal cancer.

HAP was superior to KBP and manual planning in OAR sparing, except for the lung V5. One possible explanation to the higher V5 is the better CI and the lower doses to other OARs, as there are often trade-offs between objectives. Furthermore, CI appeared in HAP group owing to the built-in target conformity management.

These present findings are similar to the findings reported by Li, Wang [3], in which automated planning was compared with manual planning in lower esophageal cancer, and it was found that automated planning resulted in more conformity and better OAR protection than manual planning, except for lung V5. However, the IMRT parameters chosen by Li, Wang [3] were based on their subjective opinions of optimal values, which may have certain impacts on the results.

Compared to full manual planning, KBP did not improve plan quality. This was because although KBP is a powerful tool for gathering information from existing treatment plans based on patient-specific anatomies and prescription information, it’s performance is limited by the quality of data set used to generate the model as well as the robustness of the modeling algorithm. In contrast, Auto-Planning employs a totally different mechanism which is more generic and protocol-specific. It’s progressive optimization algorithm, auto-generated planning structures and self-adjusted objectives enhance a planner’s abilities to manipulate dose distribution and to spare OARs. It is not surprising that HAP, a solution inherited the merits from both KBP and Auto-Planning, outperformed KBP-only in many aspects. It would be interesting to utilize HAP to build a iterative loop for optimizing KBP models, and to find how it will affect KBP and HAP.

The oncologist who participated in this study was in favor of HAP plans, as the correlation between lung V5 and pulmonary toxicity is considered not as strong as V20 or mean lung dose (MLD) [14], and based on the findings of a recent phase 3 clinical study, prioritizing lung V20 and MLD over lung V5 is recommended [15]. The better target conformity and lower heart dose would likely benefit patients in a long term. That being said, HAP plans are at least as good as KBP and manual plans from clinical perspective.

There were some limitations in the present study. First, the KBP model may not be optimal. A better KBP model may exhibit better performance in DVH prediction, thus outperform manual planning. Second, the number and direction of fields may have a considerable impact on the dose distribution, which were not taken into account in the present study. A further limitation of this study is the quality of manual plans is dependent on the experience of the planner. Future investigations on how will KPB model quality or planner’s level of experience impact the effectiveness of HAP are encouraged.

## Conclusion

The present study presents a HAP solution for esophageal cancer, which combines KBP and script-based planning. This approach is effective and recommended for esophageal cancer.

## Data Availability

The datasets used and/or analyzed during the current study are available from the corresponding author on reasonable request.

## Abbreviations

3DCRT: 3D conformal radiation therapy
CI: Conformity index
CT: Computed tomography
CTV: Clinical target volume
DVH: Dose–volume histogram
HAP: Hybrid automated treatment planning
HI: Homogeneity index
IMRT: Intensity modulated radiation therapy
KBP: Knowledge-based planning
OAR: Organs at risk
PTV: Planning target volume
VMAT: Volumetric modulated arc therapy
